# Lipid alterations in human frontal cortex in ALS‐FTLD‐TDP43 proteinopathy spectrum are partly related to peroxisome impairment

**DOI:** 10.1111/nan.12681

**Published:** 2021-01-12

**Authors:** Pol Andrés‐Benito, Ellen Gelpi, Mariona Jové, Natalia Mota‐Martorell, Èlia Obis, Manuel Portero‐Otin, Mònica Povedano, Aurora Pujol, Reinald Pamplona, Isidro Ferrer

**Affiliations:** ^1^ Neuropathology Bellvitge University Hospital‐Bellvitge Biomedical Research Institute (IDIBELL Hospitalet de Llobregat, Barcelona Spain; ^2^ Department of Pathology and Experimental Therapeutics University of Barcelona Barcelona Spain; ^3^ CIBERNED (Network Centre of Biomedical Research of Neurodegenerative Diseases Institute of Health Carlos III Ministry of Economy and Competitiveness Madrid Spain; ^4^ International Initiative for Treatment and Research Initiative to Cure ALS (TRICALS Utrecht The Netherlands; ^5^ Neurological Tissue Bank of the Biobanc‐Hospital Clínic‐Institut d'Investigacions Biomèdiques August Pi I Sunyer (IDIBAPS Barcelona Spain; ^6^ Institute of Neurology Medical University of Vienna Vienna Austria; ^7^ Department of Experimental Medicine University of Lleida ‐ Lleida Biomedical Research Institute (UdL‐IRBLleida Lleida Spain; ^8^ Functional Unit of Amyotrophic Lateral Sclerosis (UFELA Service of Neurology Bellvitge University Hospital Hospitalet de Llobregat Spain; ^9^ Catalan Institution for Research and Advanced Studies (ICREA Barcelona Spain; ^10^ Neurometabolic Diseases Laboratory Bellvitge Biomedical Research Institute Hospital Duran i Reynals Hospitalet de Llobregat, Barcelona Spain; ^11^ Center for Biomedical Research on Rare Diseases (CIBERER Institute of Health Carlos III Madrid Spain; ^12^ Institute of Neurosciences University of Barcelona Barcelona Spain

**Keywords:** fatty acid profiling, human frontal cortex, lipidomics, peroxisomes, plasmalogens, transcriptomics

## Abstract

**Aim:**

Peroxisomes play a key role in lipid metabolism, and peroxisome defects have been associated with neurodegenerative diseases such as X‐adrenoleukodystrophy and Alzheimer's disease. This study aims to elucidate the contribution of peroxisomes in lipid alterations of area 8 of the frontal cortex in the spectrum of TDP43‐proteinopathies. Cases of frontotemporal lobar degeneration‐TDP43 (FTLD‐TDP), manifested as sporadic (sFTLD‐TDP) or linked to mutations in various genes including expansions of the non‐coding region of *C9ORF72* (c9FTLD), and of sporadic amyotrophic lateral sclerosis (sALS) as the most common TDP43 proteinopathies, were analysed.

**Methods:**

We used transcriptomics and lipidomics methods to define the steady‐state levels of gene expression and lipid profiles.

**Results:**

Our results show alterations in gene expression of some components of peroxisomes and related lipid pathways in frontal cortex area 8 in sALS, sFTLD‐TDP and c9FTLD. Additionally, we identify a lipidomic pattern associated with the ALS‐FTLD‐TDP43 proteinopathy spectrum, notably characterised by down‐regulation of ether lipids and acylcarnitine among other lipid species, as well as alterations in the lipidome of each phenotype of TDP43 proteinopathy, which reveals commonalities and disease‐dependent differences in lipid composition.

**Conclusion:**

Globally, lipid alterations in the human frontal cortex of the ALS‐FTLD‐TDP43 proteinopathy spectrum, which involve cell membrane composition and signalling, vulnerability against cellular stress and possible glucose metabolism, are partly related to peroxisome impairment.

## Graphical abstract

3lipid alterations in human frontal cortex in ALS‐FTLD‐TDP43 proteinopathy spectrum are partly related to peroxisome impairment.

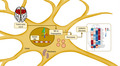

## INTRODUCTION

Peroxisomes are single membrane‐bound cytoplasmic organelles in eukaryotic cells which harbour a variety of biochemical reactions and metabolic pathways involved in oxidative stress homeostasis, and carbohydrate, amino acid and lipid metabolism. In the lipid field, peroxisomes play a key role in α‐oxidation of branched‐chain fatty acids and β‐oxidation of very long fatty acids (>C20), as well as biosynthesis of ether lipids, bile acids and docosahexaenoic acid [1, 2, 3, 4, 5, 6, 7, 8, 9]. Peroxisomes are found ubiquitously, but their number, shape and enzymatic content respond rapidly to cellular and environmental factors [6, 8, 10, 11]. Peroxisomes have close functional relationships with the endoplasmic reticulum, mitochondria and lipid droplets [12, 13, 14, 15, 16, 17]. The biogenesis of peroxisomes requires a group of proteins named peroxins, encoded by *PEX* genes, which participate in the formation of peroxisomal membranes incorporating peroxisomal membrane proteins, peroxisome proliferator‐activated receptors (encoded by *PPARS*) that modulate peroxisomal biogenesis and regulate lipid metabolism, and dynamin‐related proteins [6, 7, 18, 19, 20, 21].

Primary peroxisomal defects of lipid metabolism are genetically determined disorders linked to mutations of specific peroxisomal genes that lead to distinct diseases with neurologic and systemic manifestations and invariably poor outcomes. These may affect fatty acid β‐oxidation, ether lipid biosynthesis and fatty acid α‐oxidation [11, 31]. In addition, peroxisomes contribute to cellular ageing and redox balance, under the control of peroxisome/mitochondria function, which are altered in age‐related diseases such as diabetes, hypertension, cancer and neurodegenerative diseases [32, 33, 34, 35]. Impaired peroxisomal function occurs in Alzheimer's disease (AD) and related transgenic mouse models [36, 37, 38]. More precisely, accumulation of C22:0 and very long‐chain fatty acids, and decreased levels of plasmalogens, together with increased volume density and loss of peroxisomes in neurons with neurofibrillary tangles, are all observed with AD progression [39]. These alterations have prompted the study of several specific therapeutic tools directed to curbing altered peroxisomal function in AD [40, 41, 42, 43, 44, 45].

Amyotrophic lateral sclerosis (ALS) and frontotemporal lobar degeneration (FTLD) are two fatal neurodegenerative disorders with considerable clinical, pathological and genetic overlap. ALS is a fatal neurodegenerative disorder characterised by the progressive degeneration of both upper and lower motor neurons, resulting in a multitude of motor symptoms, including muscle weakness, fasciculations, spasticity, dysphagia and, eventually, respiratory dysfunction [46]. FTLD is a pathological diagnosis that manifests clinically in the form of frontotemporal dementia (FTD), characterised by cognitive, behavioural and linguistic dysfunction. The link between these disorders is made clear by the fact that almost 50% of ALS patients show cognitive impairment of the type observed in FTD, and also that 15% of ALS cases meet the diagnostic criteria for FTD at the time ALS is diagnosed [47]. In addition, 15% of FTLD cases have clinically detectable motor symptoms [48]. Both disorders are characterised by the accumulation of pathological protein aggregates that contain a number of proteins, most notably TAR DNA‐binding protein 43 kDa (TDP‐43).

This study is aimed at elucidating the contribution of peroxisomal alterations to lipid metabolism in frontal cortex area 8 within the spectrum of TDP‐43 proteinopathies. FTLD‐TDP, manifested as sporadic (sFTLD‐TDP) or linked to mutations in various genes including expansions of the non‐coding region of *C9ORF72* (c9FTLD), as well as sporadic ALS (sALS), are the most common TDP‐43 proteinopathies. To this end, we used transcriptomic and lipidomic methods to define the steady‐state levels of gene expression and lipid profiles. Our results show alterations in gene expression of some components of peroxisomes and related lipid pathways in frontal cortex (FC) area 8 in sALS, sFTLD‐TDP and c9FTLD.

## MATERIALS AND METHODS

### Human cases

Post‐mortem samples of fresh‐frozen FC area 8 were obtained from the Institute of Neuropathology HUB‐ICO‐IDIBELL Biobank and the Hospital Clinic‐IDIBAPS Biobank following the guidelines of Spanish legislation on this matter and approval of the local ethics committees and in accordance with criteria of sample quality [49, 50, 51]. The post‐mortem delay varied from 2 h 15 min to 18 h. This post‐mortem delay does not compromise the quality of the sample [49, 50, 51]. One hemisphere was immediately cut in coronal sections, 1 cm thick, and selected areas of the encephalon were rapidly dissected, frozen on metal plates over dry ice, placed in individual air‐tight plastic bags and stored at −80°C until the use for biochemical studies. The other hemisphere was fixed by immersion in 4% buffered formalin for 3 weeks for morphological studies.

The neuropathological study was carried out on 20 selected 4‐μm‐thick de‐waxed paraffin sections of representative regions of the brain. Sections were stained with haematoxylin and eosin, Klüver‐Barrera, or processed for immunohistochemistry with anti‐β‐amyloid, phospho‐tau (clone AT8), α‐synuclein, αB‐crystallin, TDP‐43, ubiquitin, p62, glial fibrillary acidic protein, CD68 and Iba1 antibodies [52]. Sporadic FTLD‐TDP (sFTLD‐TDP) cases were diagnosed following well‐established criteria: frontotemporal atrophy, loss of neurons and variable spongiosis in the upper cortical layers, astrocytic gliosis and presence of TDP‐43‐immunoreactive inclusions in the cytoplasm or in the nucleus of neurons, and in dendrites (NCIs, NIIs and DNs respectively), and were then categorised as type A, B or C.[53, 54] Cases with familial frontotemporal lobar degeneration linked to C9ORF72 expansion (henceforth referred to as c9FTLD for practical purposes), all of them carrying more than 30 intronic hexanucleotide repeats, were classified as type A or B. All these cases showed a sequential pattern II or III [55]. The frontal cortex of sporadic ALS (sALS) cases showed variable alterations; TDP‐43‐immunoreactive small dystrophic neurites and/or TDP‐43‐positive granules and/or small cytoplasmic globules in neurons were observed in 11 of 18 cases, but they were abundant in only three cases (cases 56, 57 and 58) (Table 1); spongiosis in the upper cortical layers was found in only one case (case 55). The whole series included 16 sFTLD‐TDP (71.6 ± 9.6 years; 11 men and 3 women), 19 c9FTLD (mean age 70 years; 10 men and 9 women), 15 sALS (mean age 54 years; 11 men and 4 women) and 17 control cases (64.7 ± 8.9 years; 11 men and 6 women), as summarised in Table 1. Although there are significant differences in the average age of the groups analysed, the age range of the study subjects is between 60 and 70 years. Previous studies on brain (and particularly in frontal cortex) lipid composition showed that lipids remain stable in adulthood; minimal changes appear in older ages than those analysed here [56, 57].

**TABLE 1:** Summary of cases

Case	Sex	Age	Diagnosis	PMD	RIN	TDP**‐**43
1	M	66	Control	18 h 00 min	6.4	–
2	M	61	Control	03 h 40 min	7.0	–
3	M	62	Control	05 h 45 min	5.0	–
4	M	74	Control	06 h 40 min	7.2	–
5	M	65	Control	05 h 15 min	6.8	–
6	F	64	Control	02 h 15 min	5.0	–
7	M	63	Control	08 h 05 min	7.1	–
8	F	79	Control	03 h 35 min	6.8	–
9	F	67	Control	05 h 20 min	6.2	–
10	M	70	Control	03 h 45 min	7.2	–
11	M	52	Control	04 h 40 min	7.2	–
12	F	52	Control	05 h 45 min	5.1	–
13	F	82	Control	07 h 35 min	5.2	–
14	F	74	Control	02 h 45 min	5.7	–
15	M	55	Control	05 h 40 min	7.7	–
16	M	59	Control	07 h 05 min	7.8	–
17	M	56	Control	03 h 50 min	7.6	–
18	M	76	sFTLD‐TDP	05 h 00 min	6.2	A
19	F	82	sFTLD‐TDP	03 h 40 min	6.4	A
20	M	71	sFTLD‐TDP	04 h 00 min	6.1	A
21	F	77	sFTLD‐TDP	16 h 00 min	6.9	C
22	M	73	sFTLD‐TDP	05 h 00 min	6.7	C
23	M	63	sFTLD‐TDP	09 h 30 min	5.0	A
24	F	77	sFTLD‐TDP	07 h 39 min	7.0	A
25	M	65	sFTLD‐TDP	13 h 00 min	7.4	A
26	F	88	sFTLD‐TDP	06 h 30 min	5.4	A
27	M	59	sFTLD‐TDP	08 h 00 min	7.4	A
28	M	58	sFTLD‐TDP	04 h 00 min	7.3	A
29	M	56	sFTLD‐TDP	08 h 00 min	5.0	A
30	F	84	sFTLD‐TDP	06 h 00 min	5.9	B
31	M	78	sFTLD‐TDP	07 h 15 min	6.7	C
32	M	66	sFTLD‐TDP	05 h 15 min	7.2	A
33	M	74	sFTLD‐TDP	15 h 00 min	6.4	C
34	M	69	c9FTLD	11 h 30 min	6.5	A‐B
35	F	69	c9FTLD	13 h 15 min	5.4	A‐B
36	M	68	c9FTLD	02 h 30 min	6.8	A‐B
37	M	61	c9FTLD	07 h 45 min	6.9	A‐B
38	M	66	c9FTLD	15 h 15 min	7.9	A‐B
39	F	55	c9FTLD	03 h 15 min	8.7	A‐B
40	M	69	c9FTLD	05 h 00 min	6.1	A‐B
41	F	75	c9FTLD	17 h 30 min	7.5	A‐B
42	F	92	c9FTLD	09 h 15 min	7.1	A‐B
43	F	58	c9FTLD	11 h 00 min	8.4	A‐B
44	F	66	c9FTLD	11 h 30 min	8.1	A‐B
45	M	73	c9FTLD	15 h 30 min	6.2	A‐B
46	F	69	c9FTLD	12 h 30 min	5.9	A‐B
47	F	57	c9FTLD	03 h 40 min	7.2	A‐B
48	M	80	c9FTLD	12 h 00 min	8.0	A‐B
49	F	57	c9FTLD	08 h 00 min	6.9	A‐B
50	M	88	c9FTLD	05 h 00 min	7.3	A‐B
51	M	69	c9FTLD	05 h 45 min	7.1	A‐B
52	M	80	c9FTLD	08 h 30 min	6.5	A‐B
53	M	70	sALS	03 h 00 min	7.0	–
54	F	56	sALS	03 h 45 min	7.7	–
55	M	59	sALS	03 h 15 min	7.7	–
56	F	63	sALS	13 h 50 min	8.2	–
57	F	59	sALS	14 h 15 min	6.7	–
58	M	54	sALS	04 h 50 min	7.8	–
59	M	76	sALS	12 h 40 min	7.4	–
60	M	64	sALS	16 h 30 min	7.3	–
61	F	57	sALS	04 h 00 min	8.6	–
62	F	75	sALS	04 h 05 min	6.8	–
63	F	57	sALS	10 h 00 min	7.1	–
64	M	50	sALS	10 h 10 min	5.9	–
65	F	59	sALS	02 h 30 min	7.5	–
66	M	46	sALS	07 h 00 min	8.0	–
67	F	69	sALS	17 h 00 min	6.3	–

Sixty‐seven cases corresponding to 17 controls, 16 sFTLD‐TDP cases, 19 c9FTLD and 15 sALS were used for this study.

A, B, C, Classification of FTLD according to [53]; F, female; M, male; PMD, post‐mortem delay (hours, minutes); RIN, RNA integrity number; TDP‐43, TDP‐43 abnormal inclusions in neurons and dendrites in frontal cortex.

Patients with additional associated pathologies of the nervous system, excepting early stages of neurofibrillary tangle pathology in the entorhinal cortex and hippocampus, and those with the presence of mild small blood vessel disease, were excluded, as were those cases with infectious, inflammatory or autoimmune diseases. Clinically, patients with FTLD had suffered from variable cognitive deficits, reporting parkinsonism and progressive aphasia in some cases. sALS cases had no cognitive alterations. Age‐matched control cases had not suffered from neurologic or psychiatric disorders, or systemic diseases, and had no neuropathological lesions other than those permitted in included disease cases.

### RNA extraction and RT**‐**qPCR validation

RNA from frozen FC area 8 was extracted following the instructions of the supplier (RNeasy Mini Kit, Qiagen® GmbH, Hilden, Germany). RNA integrity and 28S/18S ratios were determined with the Agilent Bioanalyzer (Agilent Technologies Inc, Santa Clara, CA, USA) to assess RNA quality, and the RNA concentration was evaluated using a NanoDrop™ Spectrophotometer (Thermo Fisher Scientific). Complementary DNA (cDNA) preparation used a High‐Capacity cDNA Reverse Transcription kit (Applied Biosystems, Foster City, CA, USA) following the protocol provided by the supplier. TaqMan RT‐qPCR assays were performed in duplicate for each gene on cDNA samples in 384‐well optical plates using an ABI Prism 7900 Sequence Detection system (Applied Biosystems, Life Technologies, Waltham, MA, USA). For each 10 μl TaqMan reaction, 2.25 μl cDNA was mixed with 0.25 μl 20× TaqMan Gene Expression Assays and 2.50 μl of 2× TaqMan Universal PCR Master Mix (Applied Biosystems). The identification numbers and names of TaqMan probes are shown in Table S1. The values for β‐glucuronidase (GUS‐β) were used as internal controls for normalisation purposes [58]. The parameters of the reactions were 50°C for 2 min, 95°C for 10 min and 40 cycles of 95°C for 15 s and 60°C for 1 min. Finally, Sequence Detection Software (SDS version 2.2.2, Applied Biosystems) was used to capture TaqMan PCR data. The double‐delta cycle threshold (ΔΔCT) method was utilised to analyse the data. The statistical study was performed using the T‐student test or ANOVA‐one way when necessary. The significance level was set at *p < 0.05, **p < 0.01 and ***p < 0.001 versus control group; ^#^ *p* < 0.05, ^##^ *p* < 0.01 and ^###^ *p* < 0.001 versus sALS; and ^$^ *p* < 0.05, ^$$^ *p* < 0.01 and ^$$$^ *p* < 0.001 versus sFTLD‐TDP.

### Fatty acid profiling

Briefly, samples were incubated for lipid extraction and FAs transesterification in 2 ml of 5% methanolic HCL at 75 °C for 90 min. FAs methyl esters were extracted by adding 2 ml of n‐pentane and 1 ml of saturated NaCl solution. Samples were separated and evaporated under N2 gas n‐pentane phase and finally dissolved in 80 µl of carbon disulphide. Gas chromatography (GC) analysis was then performed.

The GC method was used for separation with a DBWAX capillary column (30 m × 0.25 mm ×0.20 μm) in a GC System 7890 A with a Series Injector 7683B and an FID detector (Agilent Technologies, Barcelona, Spain). The temperature of the injector was 220 °C using the splitless mode. A constant rate (1.8 ml/min) of helium (99.99%) was maintained. The column temperature was held at 145°C for 5 min; subsequently, the column temperature was increased by 2°C/min to 245°C for 50 min, and held at 245°C for 10 min, with a post‐run of 250°C for 10 min as previously described [59, 60, 61]. Based on FA composition, different indexes were calculated, and elongase and desaturase activity was estimated from specific product/substrate ratios [61, 62].

### Non**‐**targeted lipidomic analysis

A previously validated method was used for lipid extraction [63]. Briefly, 5 μl of miliQ water and 20 μl of methanol were added to 10 μl of homogenised tissue. Samples were then shaken vigorously for 2 min. Following this, methyl tert‐butyl ether (MTBE) containing isotopically labelled lipid standards was added. Samples were then immersed in a water bath (ATU Ultrasonidos, Valencia, Spain) with an ultrasound frequency of 40 kHz and power of 100 W, at 10°C for 30 min. After this, 25  μl of miliQ water was added to the mixture, which was centrifuged at 300 rpm at 4°C for 10 min to separate the organic phase. Finally, the upper phase was collected and stored for mass‐spectrometry analysis. A pool (20 µl of each sample) of all lipid extracts was prepared and used as quality control [64].

Lipid extracts were analysed by LC‐MS according to the method described [59]. An Agilent UPLC 1290 system coupled to an ESI‐Q‐TOF MS/MS 6545 (Agilent Technologies, Barcelona, Spain) was used. Two runs were performed to collect positive and negative electrospray ionised lipid species. Data pre‐processing was done as published before [65, 66, 67]. Finally, identities were confirmed by searching experimental MS/MS spectra against in silico libraries, using HMDB and LipidMatch, an R‐based tool for lipid identification [68, 69]. Multivariate and univariate statistics were calculated using Metaboanalyst [70].

## RESULTS

### Peroxisome**‐**related genes

#### Peroxisome biogenesis

**FIGURE 1:**
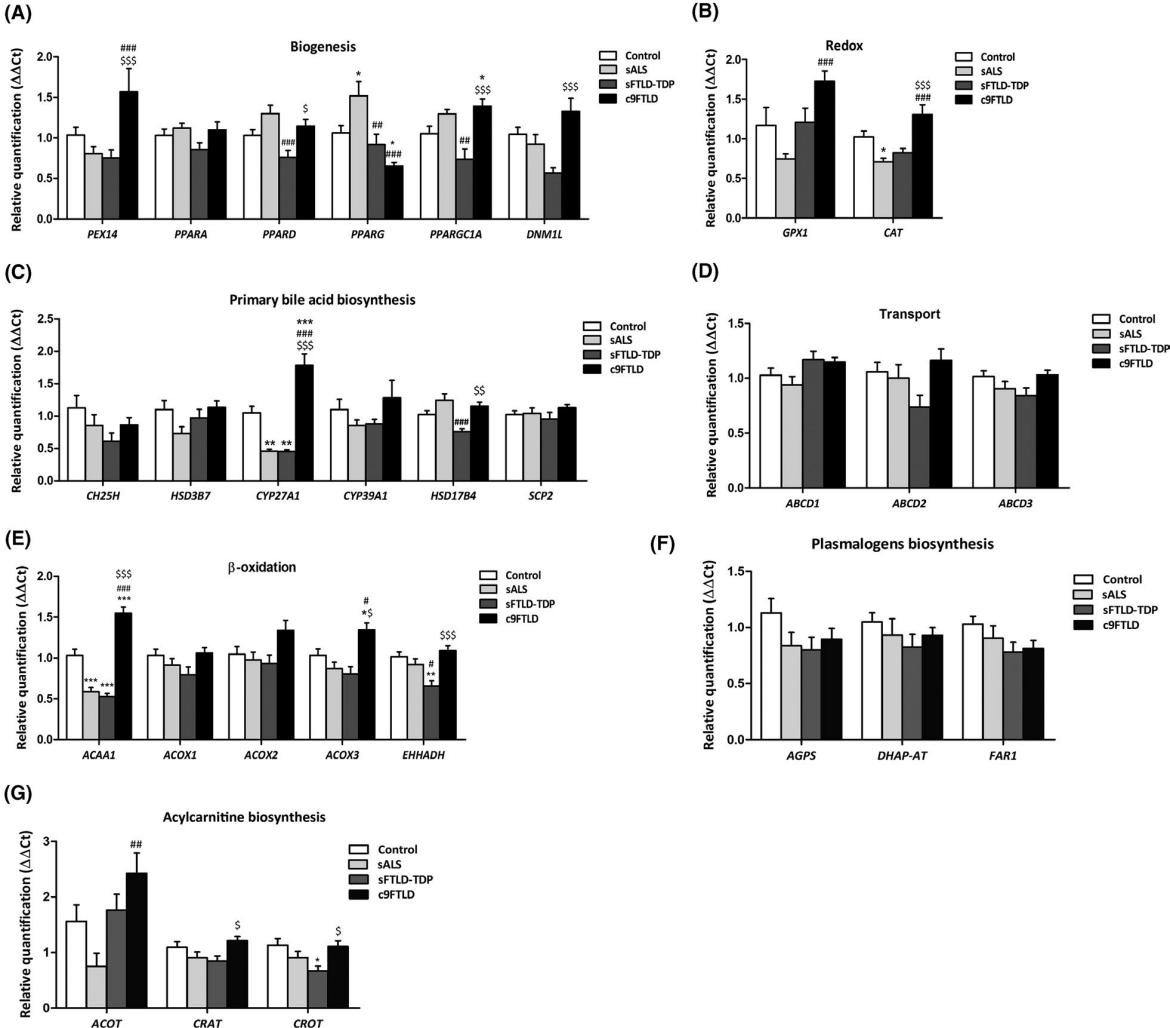
mRNA expression levels of peroxisome‐related genes in frontal cortex area 8 in controls, sALS, sFTLD‐TDP and c9FTLD cases assessed with TaqMan RT‐qPCR assays. (A) Genes implicated in peroxisome biogenesis. (B) Genes coding for redox mechanisms. (C) Genes involved in peroxisomal primary bile acid biosynthesis. (D) Genes related with peroxisome substrate transport. (E) Genes involved in peroxisomal β‐oxidation. Genes linked to plasmalogens biosynthesis (F) and acylcarnitine biosynthesis (G). Data are expressed as the mean values±SEM. The significance level was set at *p < 0.05, **p < 0.01 and ***p < 0.001 versus control group; ^#^ *p* < 0.05, ^##^ *p* < 0.01 and ^###^ *p* < 0.001 versus sALS; and ^$^ *p* < 0.05, ^$$^ *p* < 0.01 and ^$$$^ *p* < 0.001 versus sFTLD‐TDP

When compared with controls, only *PPARG* expression was significantly increased in sALS (*p* < 0.01) and significantly decreased in c9FTLD cases (*p* = 0.018), whereas *PPARGC1A* was significantly increased in c9FTLD (*p* = 0.033). However, differences were also identified when comparing expression levels among the three disease groups. Thus, *PEX14* was significantly increased in c9FTLD when compared with sALS (*p* < 0.001) and sFTLD‐TDP (*p* < 0.001); *PPARD* was decreased in sFTLD‐TDP when compared with sALS (*p* < 0.001); *PPARG* was decreased in sFTLD‐TDP and c9FTLD when compared with sALS (*p* = 0.006 and *p* < 0.001 respectively); *PPARGC1A* was down‐regulated in sFLTD‐TDP when compared with sALS (*p* < 0.005) and up‐regulated in c9FTLD when compared with sFTLD‐TDP (*p* < 0.001); and finally, *DNM1L* mRNA expression was significantly increased in c9FTLD when compared with sFTLD‐TDP (*p* < 0.001). No differences were detected for PPARA. See Figure 1A.

#### Peroxisome redox mechanisms

*GPX1* mRNA levels were increased in c9FTLD when compared with sALS (*p* < 0.001), whereas *CAT* transcript levels were down‐regulated in sALS when compared with controls (*p* = 0.011), and up‐regulated in c9FTLD when compared with sALS and sFTLD‐TDP (*p* < 0.001 and *p* < 0.001 respectively) (Figure 1B).

#### Primary bile acid metabolism

*CYP27A1* mRNA levels were down‐regulated in sALS and sFTLD‐TDP when compared with controls (*p* = 0.002 and *p* = 0.006 respectively). In contrast, *CYP27A1* gene expression was up‐regulated in c9FTLD when compared with controls, sALS and FTLD‐TDP (*p* < 0.001). Reduced *HSD17B4* expression was limited to sFTLD‐TDP when compared with sALS and c9FTLD (*p* = 0.001, and *p* = 0.006 respectively). No differences in the expression of CH25H, HSD3B7, CYP39A1 or SCP2 were found between controls and disease cases, nor among the three pathological groups (Figure 1C).

#### Transport

No modifications in the expression of genes coding for β‐oxidation ATP‐binding cassette (ABC) transporters *ABCD1*, *ABCD2* and *ABCD3* were observed in sALS, sFTLD‐TDP or c9FTLD when compared with controls. Nor were significant differences observed among the three disease groups (Figure 1D).

#### β**‐**oxidation components

*ACAA1* gene expression was decreased in sALS and sFTLD‐TDP when compared with controls (*p* < 0.001 and *p* < 0.001 respectively), but it was increased in c9FTLD, not only when compared with controls (*p* < 0.001), but also with respect to sALS (*p* < 0.001) and sFTLD‐TDP cases (*p* < 0.001). Following a similar trend, levels of *ACOX3* were increased in c9FTLD when compared with controls (*p* < 0.001), sALS (*p* < 0.001) and sFTLD‐TDP (*p* < 0.001). *EHHADH* transcript levels were down‐regulated in sFTLD‐TDP when compared with controls and sALS (*p* = 0.004 and *p* = 0.049 respectively), and up‐regulated in c9FTLD when compared with sFTLD‐TDP (*p* < 0.001). No differences in the expression of ACOX1 and ACOX2 were found between controls and disease cases, nor among the three pathological groups (Figure 1E).

#### Plasmalogen biosynthesis

Expression levels of genes coding for components of the plasmalogen biosynthesis pathway, *AGPS*, *DHAP*‐*AT* and *FAR1*, were evaluated, but no differences in the expression of these genes were found between controls and disease cases, or among the three pathological groups (Figure 1F).

#### Acylcarnitine biosynthesis

Acylcarnitine biosynthesis components revealed few differences in the expression of *ACOT*, *CRAT* and *CROT* transcripts. *ACOT* gene expression was increased in c9FTLD when compared with sALS (*p* = 0.003), whereas *CRAT* and *CROT* were significantly increased in c9FTLD when compared with sFTLD cases (*p* = 0.034 and *p* = 0.029 respectively). Regarding control cases, only *CROT* mRNA expression levels were significantly decreased in sFTLD cases when compared to controls (*p* = 0.021) (Figure 1G).

### Gene expression linked to fatty acid metabolism

**FIGURE 2:**
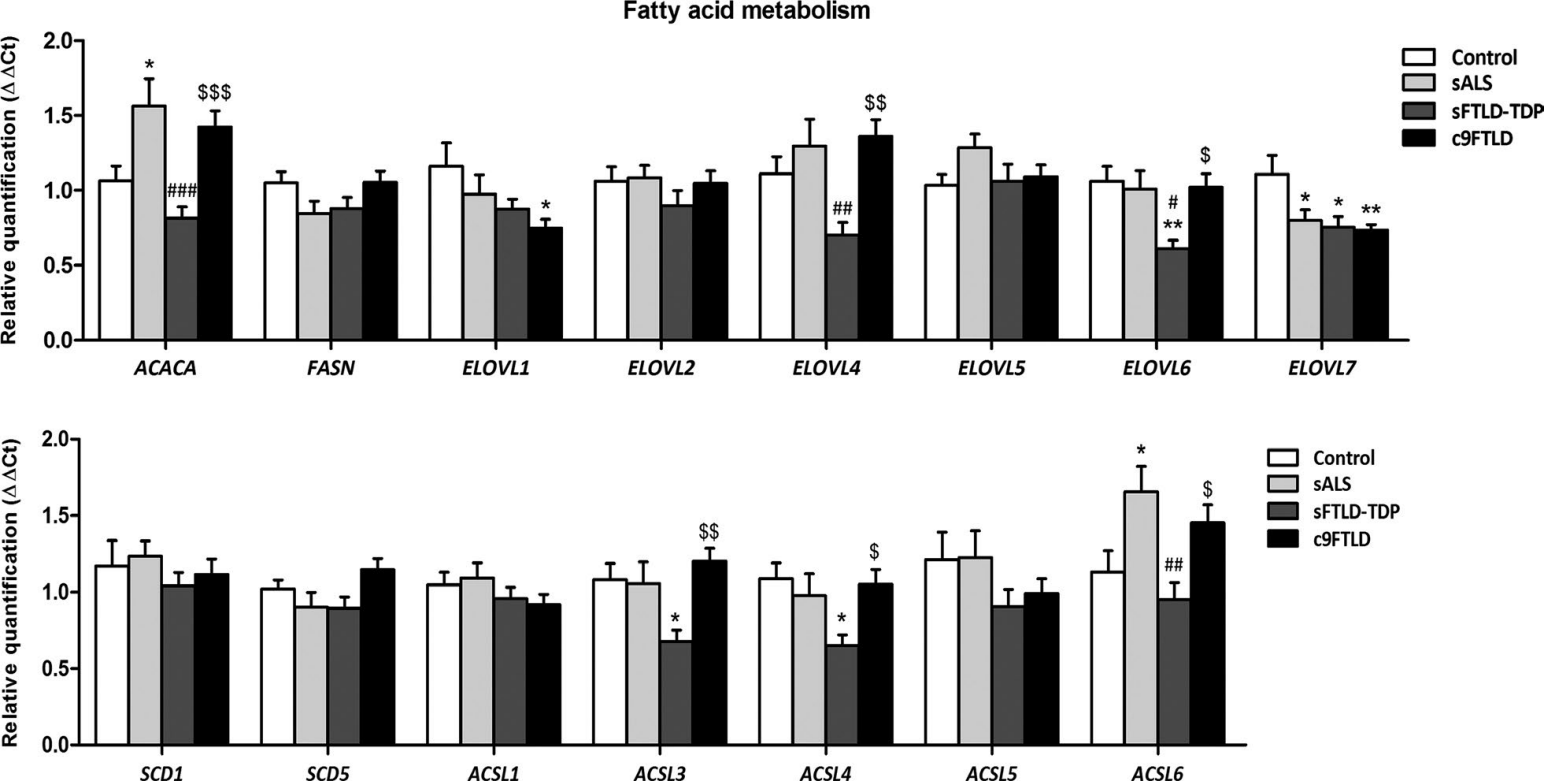
mRNA expression levels of genes linked to fatty acid metabolism in frontal cortex area 8 in controls, sALS, sFTLD‐TDP and c9FTLD cases assessed with TaqMan RT‐qPCR assays. Data are expressed as the mean values±SEM. The significance level was set at *p < 0.05, **p < 0.01 and ***p < 0.001 versus control group; ^#^ *p* < 0.05, ^##^ *p* < 0.01 and ^###^ *p* < 0.001 versus sALS; and ^$^ *p* < 0.05, ^$$^ *p* < 0.01 and ^$$$^ *p* < 0.001 versus sFTLD‐TDP

The expression of fifteen genes was assessed. No modifications in the mRNA expression levels of *FASN*, *ELOVL2*, *ELOVL5*, *SCD1*, *SCD5*, *ACSL1* or *ACSL5* were identified in the three diseases when compared with controls, nor among the pathological groups. In sALS, only *ACACA* and *ACSL6* were significantly increased, and *ELOVL7* significantly decreased when compared with controls (*p* = 0.026, *p* = 0.04 and *p* = 0.05 respectively). Regarding sFTLD‐TDP, *ELOVL6*, *ELOVL7*, *ACSL3* and *ACSL4* were significantly decreased when compared with controls (*p* = 0.04, *p* = 0.028, *p* = 0.031 and *p* = 0.028 respectively). In addition, *ACACA*, *ELOVL4*, *ELOVL6* and *ACSL6* were significantly decreased in sFTLD‐TDP when compared with sALS (*p* < 0.001, *p* = 0.009, *p* = 0.032 and *p* = 0.003 respectively). Finally, expression levels of *ELOVL1* and *ELOVL7* mRNA were significantly decreased in c9FTLD when compared with controls (*p* = 0.01, and *p* = 0.041 respectively), but *ACACA*, *ELOVL4*, *ELOVL6*, *ACSL3*, *ACSL4* and *ACSL6* significantly increased in c9FTLD when compared with sFTLD‐TDP (*p* = 0.001 *p* = 0.002, *p* = 0.018, *p* = 0.002, *p* = 0.018 and *p* = 0.038 respectively) (Figure 2).

### Fatty acid profiling

Since the biosynthesis of highly unsaturated fatty acids is dependent on peroxisomal beta‐oxidation activity, fatty acid composition of total lipids from frontal cortex area 8 was analysed (Table 2). This analysis showed no significant changes in fatty acid profiles among the FTLD‐TDP/ALS spectrum pathologies. Notably, the content of 22:5n6 and 22:6n3, highly PUFAs resulting from peroxisomal beta‐oxidation, was sustained among the FTLD‐TDP/ALS spectrum at equivalent levels as observed in the control group. As a result of this, the total number of double bonds and the global susceptibility to peroxidation, estimated as the global fatty acid indexes DBI and PI, as well as other indexes such as average chain length, SFA and UFA content, were not modified by the pathological condition, nor were desaturase and elongase activities calculated from fatty acid profiles. Only minor changes were observed for total PUFAn6 content (*p* = 0.039) and Elovl 2 (n3) estimated activity. Specifically, statistically significant differences were found in total PUFAn‐6 in sFTLD‐TDP when compared with control and c9FTLD groups, and in Elovl2(n‐3) between the control and pathogenic groups.

**TABLE 2:** Fatty acid compositional profiles of total lipids from frontal cortex area 8 in controls, sALS, sFTLD‐TDP and c9FTLD cases assessed with gas chromatography

	Control	sALS	sFTLD**‐**TDP	c9FTLD	*p*
Fatty acids					
14:0	1.44 ± 0.09	1.47 ± 0.17	1.51 ± 0.08	1.54 ± 0.12	0.716
16:0	24.73 ± 1.13	24.34 ± 2.06	24.91 ± 1.55	24.04 ± 1.51	0.946
16:1n7	1.61 ± 0.15	1.87 ± 0.39	1.92 ± 0.22	2.02 ± 0.23	0.643
18:0	24.8 ± 0.53	28.28 ± 2.62	24.3 ± 0.54	23.52 ± 0.55	0.05
18:1n9	21.77 ± 1.41	19.8 ± 3.05	23.07 ± 1.84	23.89 ± 2	0.586
18:1n7	4.58 ± 0.21	3.63 ± 0.63	4.94 ± 0.19	4.72 ± 0.19	0.105
18:2n6	0.77 ± 0.12	0.7 ± 0.13	0.61 ± 0.05	0.59 ± 0.02	0.475
18:3n3	0.07 ± 0.01	0.08 ± 0.01	0.07 ± 0.01	0.08 ± 0.01	0.962
18:4n3	0.94 ± 0.06	1.18 ± 0.25	1.06 ± 0.09	0.88 ± 0.08	0.512
20:0	0.24 ± 0.00	0.3 ± 0.03	0.25 ± 0	0.24 ± 0.01	0.073
20:1n9	1.32 ± 0.17	1.93 ± 0.66	1.41 ± 0.25	1.35 ± 0.23	0.993
20:2n6	0.45 ± 0.04	0.54 ± 0.19	0.46 ± 0.08	0.42 ± 0.06	0.954
20:3n3	0.69 ± 0.03	0.67 ± 0.04	0.61 ± 0.02	0.63 ± 0.08	0.282
20:4n6	4.89 ± 0.21	5.02 ± 0.51	4.71 ± 0.32	4.49 ± 0.33	0.764
20:3n6	0.27 ± 0.05	0.17 ± 0.02	0.16 ± 0.03	0.16 ± 0.04	0.161
22:0	0.03 ± 0.001	0.03 ± 0.01	0.03 ± 0.001	0.03 ± 0.001	0.113
20:5n3	0.61 ± 0.13	0.59 ± 0.1	0.51 ± 0.04	0.42 ± 0.03	0.278
22:1	0.06 ± 0.01	0.08 ± 0.03	0.07 ± 0.01	0.07 ± 0.01	0.964
22:4n6	2.83 ± 0.19	2.58 ± 0.21	2.53 ± 0.16	2.32 ± 0.06	0.225
22:5n6	0.52 ± 0.06	0.39 ± 0.06	0.49 ± 0.09	0.40 ± 0.08	0.578
22:5n3	0.11 ± 0.01	0.14 ± 0.03	0.11 ± 0.02	0.12 ± 0.02	0.99
24:0	0.48 ± 0.07	0.51 ± 0.1	0.42 ± 0.04	0.48 ± 0.08	0.985
22:6n3	4.17 ± 0.47	3.22 ± 0.64	3.6 ± 0.46	3.33 ± 0.52	0.704
24:1n7	1.1 ± 0.22	1.41 ± 0.5	1.18 ± 0.25	1.29 ± 0.3	0.992
24:5n3	1.3 ± 0.22	0.82 ± 0.16	0.81 ± 0.13	2.74 ± 1.4	0.329
24:6n3	0.21 ± 0.05	0.26 ± 0.08	0.25 ± 0.06	0.24 ± 0.05	0.978
Fatty acid indexes					
SFA	51.71 ± 1.48	54.92 ± 2.96	51.44 ± 1.95	49.85 ± 1.89	0.533
UFA	48.29 ± 1.48	45.08 ± 2.96	48.56 ± 1.95	50.15 ± 1.89	0.513
PUFA	17.83 ± 0.65	16.36 ± 1.25	15.97 ± 0.79	16.81 ± 1.66	0.892
MUFA	30.46 ± 2.03	28.72 ± 3.71	32.59 ± 2.72	33.34 ± 2.88	0.441
PUFAn3	8.1 ± 0.54	6.97 ± 0.94	7.01 ± 0.64	8.43 ± 1.55	0.092
PUFAn6	9.73 ± 0.33	9.39 ± 0.51	8.96 ± 0.26	8.38 ± 0.35	0.039^b^, ^d^
ACL	18.13 ± 0.02	18.06 ± 0.03	18.02 ± 0.01	18.12 ± 0.1	0.964
DBI	109.63 ± 1.72	99.69 ± 4.97	103.11 ± 1.66	108.54 ± 6.55	0.691
PI	89.01 ± 4.24	78.44 ± 7.69	79.03 ± 5.25	84.98 ± 10.49	0.809
Estimated desaturase and elongase activities					
Δ9(n−7)	0.07 ± 0.01	0.09 ± 0.03	0.08 ± 0.01	0.09 ± 0.02	0.506
Δ9(n−9)	0.89 ± 0.08	0.77 ± 0.15	0.96 ± 0.1	1.03 ± 0.11	0.433
Δ5(n−6)	22.08 ± 4.98	30.13 ± 2.64	32.52 ± 3.9	42.36 ± 12.89	0.768
Δ6(n−3)	15.14 ± 2.36	18.94 ± 5.96	16.19 ± 2.51	12.78 ± 2.01	0.302
Δ6(n−3)	0.21 ± 0.07	0.52 ± 0.24	0.42 ± 0.14	0.25 ± 0.09	0.444
Elovl3(n−9)	0.06 ± 0.01	0.14 ± 0.09	0.06 ± 0.01	0.05 ± 0.01	0.928
Elovl6	1.01 ± 0.03	1.23 ± 0.2	0.99 ± 0.04	1 ± 0.05	0.997
Elovl1‐3‐7a	0.01 ± 0	0.01 ± 0	0.01 ± 0	0.01 ± 0	0.346
Elovl1‐3‐7b	0.1 ± 0.01	0.1 ± 0.02	0.14 ± 0.01	0.11 ± 0.01	0.164
Elovl1‐3‐7c	19.55 ± 3.24	29.26 ± 11.07	13.45 ± 2.66	21.54 ± 5.25	0.105
Elovl5(n−6)	0.66 ± 0.12	1.12 ± 0.5	0.79 ± 0.16	0.73 ± 0.11	0.672
Elovl2‐5 (n−6)	0.59 ± 0.05	0.56 ± 0.09	0.56 ± 0.07	0.54 ± 0.05	0.42
Elovl 2–5(n−3)	0.2 ± 0.03	0.23 ± 0.04	0.21 ± 0.03	0.29 ± 0.06	1
Elovl 2(n−3)	12.17 ± 2.04	6.38 ± 0.5	8.24 ± 1.93	36.21 ± 20.44	0.028^a^, ^b^, ^c^
Peroxisome β‐oxidation	1.54 ± 0.22	1.36 ± 0.29	1.52 ± 0.27	1.47 ± 0.24	0.691

Values are reported as mean±SEM from 6 to 8 cases and are expressed as mol%. Inter‐group differences were measured by pairwise Kruskal–Wallis test applying a Bonferroni correction. Minimum significance level is set at *p* < 0.05. Estimated desaturase and elongase activities from specific product/substrate ratios: Δ9(n−7), ratio 16:1n−9/16:0; Δ9(n−9), ratio 18:1n−9/18:0; Δ5(n−6), ratio 20:4n−6/20:3n−6; Δ6(n−3), ratio 18:4n−3/18:3n−3; Δ6(n−3), ratio 24:6n−3/24:5n−3; Elovl3(n−9), ratio 20:1n−9/18:1n−9; Elovl6, ratio 18:0/16:0; Elovl1‐3‐7a, ratio 20:0/18:0; Elovl1‐3‐7b, ratio 22:0/20:0; Elovl1‐3‐7c, ratio 24:0/22:0; Elovl5(n−6), ratio 20:2n−6/18:2n−6; Elovl2‐5 (n−6), ratio 22:4n−6/20:4n−6; Elovl 2–5(n−3), 22:5n−3/20:5n−3; Elovl 2(n−3), 24:5n−3/22:5n−3; Peroxisome β‐oxidation, ratio 22:6n−3/24:6n−3.

^a^ Control versus sALS.

^b^ Control versus sFTLD‐TDP.

^c^ Control versus c9FTLD.

^d^ sFTLD‐TDP versus c9FTLD.

### Lipidomic profiling

In order to gain an overview of whole lipidome, an untargeted lipidomic approach was applied. Baseline correction, peak picking and peak alignment were performed on acquired data, resulting in a total of 7951 molecules from both ionisation modes (negative and positive). After quality control assessment, filtering and signal correction, 1119 features remained, which were log‐transformed and auto‐scaled (mean centering/standard deviation) and used for multivariate and univariate statistical analysis.

### A lipidomic pattern is associated with ALS**‐**FTLD**‐**TDP43 proteinopathy spectrum

**FIGURE 3:**
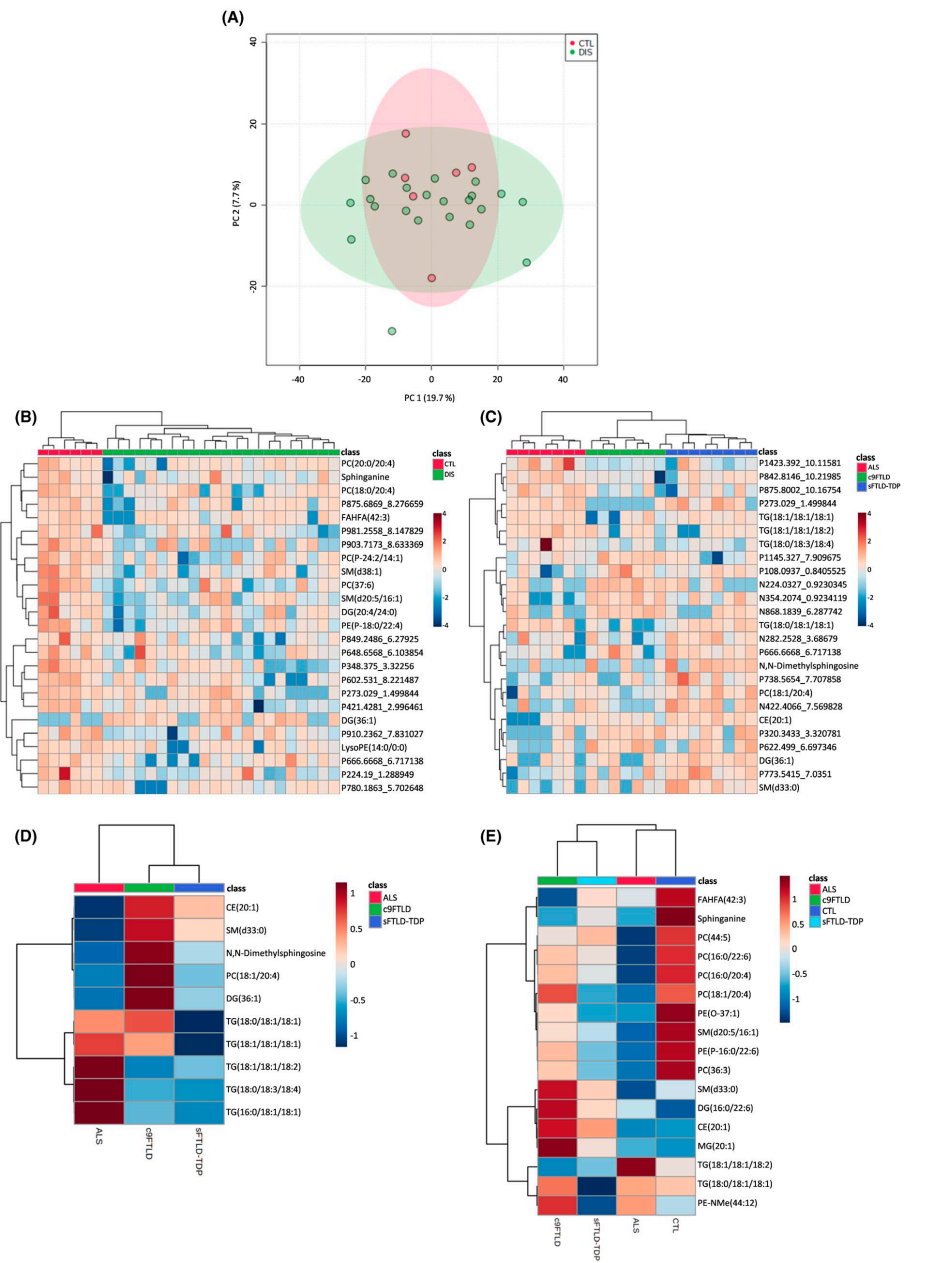
Multivariate statistics and learning methods reveal a specific lipidome shared by TDP‐43 proteinopathies. (A) Principal component analysis (PCA) 2D plot of samples lipidome. (B–C) Heatmap representation of hierarchical clustering of individual samples according to the top 25 statistically significantly different lipid species between (B) neurological disorders versus controls and (C) among groups of TDP‐43 proteinopathies. (D–E) Heatmap representation of hierarchical clustering of groups according to the identified significantly different lipid species (D) among groups of TDP‐43 proteinopathies and (E) between neurological disorders and controls

To investigate whether there was a common pattern for TDP‐43 proteinopathies, an untargeted lipidomic analysis was performed in tissue samples from frontal cortex region 8 with the three neurological disorders grouped together. Unsupervised methods such as Principal Component Analysis (PCA) and Hierarchical Clustering visualised as a heatmap were used to find patterns in the samples. A PCA analysis was performed using the whole detected lipidome; no differences were found between the diseased (DIS) group and healthy controls (CTL) (Figure 3A **)**. However, when the 25 lipid species with the lowest *p* ‐values were represented using hierarchical clustering analyses, as shown in a heatmap (Figure 3B), a clear separation between groups was observed revealing a specific shared trend in patients with neurodegenerative diseases within the ALS‐FTLD‐TDP43 proteinopathy spectrum. Finally, the Wilcoxon test on all acquired data was performed to determine whether there were any significant lipid feature differences between healthy and diseased groups (*p* < 0.05). Dunn's test was used to correct for multiple comparisons. The statistical test resulted in 63 differential molecules with *p* < 0.05 (Table 3), of which 30 were identified based on exact mass, retention time and/or MSMS spectrum.

**TABLE 3:** Lipidomic features associated with ALS‐FTLD‐TDP43 proteinopathy spectrum

Lipid category	Lipidomic feature	Ionisation	*mz* value	Retention time	*p* value	Regulation
Fatty acyls	AcCar(8:1DC)^a^	Positive	315.118	0.9	0.031093	Down
	FAHFA(42:3)^b^	Negative	643.5684	9.1	0.012598	Down
	FAHFA(5:0/22:3)^a^	Negative	433.3377	5.4	0.033121	Down
Glycerolipids	DG(20:4/24:0)^a^	Positive	746.711	7.2	0.019979	Down
	DG(36:1)^c^	Positive	640.5837	7.5	0.018745	Up
	DG(36:4)^c^	Positive	627.5308	7.3	0.031093	Down
	DG(40:6)^c^	Positive	651.5308	6.4	0.046311	Down
Glycerophospholipids	PC(16:0/22:6)^a^	Positive	806.5692	6.8	0.033121	Down
	PC(16:1/18:2)^a^	Positive	756.5511	6.7	0.021125	Down
	PC(18:0/20:4)^a^	Positive	810.5987	7.3	0.012477	Down
	PC(20:0/20:4)^a^	Positive	838.6325	7.9	0.014856	Down
	PC(37:6)^b^	Positive	784.5814	7.2	0.0097892	Down
	PC(42:7)^b^	Positive	860.6127	7.3	0.046713	Down
	PC(44:5)^b^	Positive	874.6672	8	0.021786	Down
	**PC(P‐18:1/22:5)** ^a^	**Positive**	**818.6436**	**7.8**	**0.020481**	**Down**
	PC(P**‐**24:2/14:1)^a^	Positive	796.5834	7.7	0.0052875	Down
	LysoPE(14:0/0:0)^a^	Negative	425.2602	0.9	0.010806	Down
	PE(18:0/20:4)^a^	Positive	768.5532	7.3	0.020132	Down
	PE(18:0/22:4)^a^	Positive	796.577	7.2	0.04434	Down
	PE(18:0/22:6)^a^	Positive	792.5538	7.2	0.044758	Down
	**PE(O‐37:1)** ^b^	**Positive**	**746.5681**	**7.7**	**0.0080321**	**Down**
	**PE(P‐16:0/22:6)** ^a^	**Positive**	**748.5247**	**7**	**0.024038**	**Down**
	**PE(P‐18:0/22:4)** ^a^	**Positive**	**780.5886**	**7.9**	**0.017083**	**Down**
	PG(14:1/14:1)^a^	Positive	663.4529	7.8	0.033121	Down
Sphingolipids	Cer(d18:1/17:0)^a^	Positive	548.4996	8.3	0.019979	Down
	SM(d20:5/16:1)^a^	Positive	721.5057	7.8	0.01427	Down
	SM(d38:1)^b^	Positive	759.6368	7.9	0.0077921	Down
	Sphinganine^c^	Positive	284.2891	4.4	0.017319	Down
Unknown	112.0521_0.8908437	Positive	113.0521	0.9	0.029833	Down
	1145.327_7.909675	Positive	1146.327	7.9	0.024038	Down
	1168.27_8.160657	Positive	1169.27	8.2	0.020311	Down
	1387.036_0.9344499	Positive	1388.036	0.9	0.033121	Down
	1445.068_0.9473143	Positive	1446.068	0.9	0.035663	Down
	224.19_1.288949	Positive	225.19	1.3	0.00089717	Down
	266.152_0.9234737	Negative	265.152	0.9	0.035739	Down
	268.1307_0.840175	Positive	269.1307	0.8	0.024038	Down
	273.029_1.499844	Positive	274.029	1.5	0.0073599	Down
	278.1875_0.9220529	Negative	277.1875	0.9	0.046734	Down
	311.3201_5.293459	Positive	312.3201	5.3	0.035663	Down
	322.1781_0.9135807	Positive	323.1781	0.9	0.045145	Down
	348.375_3.32256	Positive	349.375	3.3	0.0020598	Down
	377.316_1.459708	Positive	378.316	1.5	0.041782	Down
	421.4281_2.996461	Positive	422.4281	3	0.01427	Down
	488.4371_6.214781	Positive	489.4371	6.2	0.020311	Down
	598.5024_8.868424	Positive	599.5024	8.9	0.019663	Down
	602.531_8.221487	Positive	603.531	8.2	0.0032711	Down
	604.5456_7.708292	Positive	605.5456	7.7	0.033121	Down
	608.4662_2.848297	Positive	609.4662	2.8	0.020311	Down
	610.5369_7.541813	Positive	611.5369	7.5	0.034782	Down
	648.6568_6.103854	Positive	649.6568	6.1	0.01427	Down
	650.5316_7.211145	Positive	651.5316	7.2	0.033121	Down
	666.6668_6.717138	Positive	667.6668	6.7	0.014815	Down
	683.1998_8.170667	Positive	684.1998	8.2	0.044758	Down
	703.5196_6.254575	Positive	704.5196	6.3	0.033121	Down
	761.5996_8.059616	Positive	762.5996	8.1	0.020311	Down
	780.1863_5.702648	Positive	781.1863	5.7	0.0091894	Down
	849.2486_6.27925	Positive	850.2486	6.3	0.011865	Down
	856.5926_2.848731	Positive	857.5926	2.8	0.020311	Down
	875.6869_8.276659	Positive	876.6869	8.3	0.010806	Down
	891.6605_8.298165	Positive	892.6605	8.3	0.046253	Down
	903.7173_8.633369	Positive	904.7173	8.6	0.018183	Down
	910.2362_7.831027	Positive	911.2362	7.8	0.0052875	Down
	981.2558_8.147829	Positive	982.2558	8.1	0.01427	Down

Significant molecular features after Wilcoxon rank‐sum test with *p* value <0.05 are shown as LC‐ESI Ionisation mode (P for positive and N for negative) followed by neutral mass and retention time. Potential identities based on MS1 and MS2 data for each feature are found lipidomic feature. Unidentified compounds are labelled as neutral mass and retention time. Ether phospholipids are highlighted in bold.

Abbreviations: AcCar, acylcarnitine; DG, diacylglycerol; FAHFA, fatty acid ester of hydroxyl fatty acid; FDR, false discovery rate; mz value, mass‐to‐charge ratio; PC, phosphocholine; PE, phophoethanolamine; SM, sphingomyelin.

^a^ Represents confirmation by data‐dependent MS2.

^b^ Represents confirmation by data‐dependent MS2 by class.

^c^ Represents confirmation by MS1 exact mass and retention time.

Most of the identified lipids were glycerophospholipids, but a number of glycerolipids and sphingolipids were also found. Also, an acylcarnitine and two fatty acid esters of hydroxyl fatty acids were identified. Noteworthy were the phosphocholines (PC) because many of them, seven to be specific, were significantly different, with four identified at the compound level. Two of them contained 20:4 n6 (arachidonic acid, AA), one contained 22:6 n3 (docosahexaenoic acid, DHA), and one contained 18:2n6 (linoleic acid, LA). AA and DHA were also found in two of the three significantly changed phosphoethanolamines (PE). Notably, we found five ether lipids down‐regulated in the diseased samples, two of them plasmenyl phosphocholines and three plasmenyl phosphoethanolamines, again containing AA and DHA. Two sphingomyelins, one ceramide and sphinganine were identified as ALS‐FTLD‐TDP43 proteinopathy spectrum‐associated molecular features. Among the identified compounds, no bile acid was detected as different.

### Differences between neurological diseases within the ALS**‐**FTLD**‐**TDP43 proteinopathy spectrum

To determine whether there was a real difference between the distinct TDP43 proteinopathy phenotypes characterised as sALS, sFTLD‐TDP and c9FTLD, the three diseases were compared with each other. Frontal cortex region 8 samples were used to uncover characteristic lipidomic trends and features for each disorder. The heatmap representing the hierarchical clustering of the individual samples (Figure 3D) showed perfectly arranged samples in disease groups when the top differential metabolites obtained with the Kruskal–Wallis test were used. Interestingly, FTLD‐TDP patients (both sporadic and c9) clustered together, indicating that these groups are more similar to each other than they are to the ALS group.

The Kruskal–Wallis test revealed changes in glycerolipids, glycerophospholipids, sphingolipids and sterol lipids listed in Table 4. Most of the compounds identified in this analysis (Figure 3E) of frontal cortex region 8 were TGs and most contained 18:1 oleic acid; these TGs were higher in the ALS group and lower in the sFTLD‐TDP group. Interestingly, CE (20:1) was increased in c9FTLD compared with the other groups.

**TABLE 4:** Lipidomic features associated with different neurological diseases within the ALS‐FTLD‐TDP43 proteinopathy spectrum

Lipid category	Lipidomic feature	Ionisation	mz value	Retention time	chi squared	*p* value	post hoc
Glycerolipids	DG(36:1)^c^	Positive	640.5837	7.5	6.111	0.047099	sALS ‐ c9FTLD, sFTLD‐TDP ‐ c9FTLD
	TG(16:0/18:1/18:1)^a^	Positive	876.8002	10.2	6.0758	0.047935	sALS ‐ sFTLD‐TDP, sALS ‐ c9FTLD
	TG(18:0/18:1/18:1)^a^	Positive	904.8296	10.3	8.8888	0.011744	sALS ‐ sFTLD‐TDP, sFTLD‐TDP ‐ c9FTLD
	TG(18:0/18:3/18:4)^a^	Positive	894.7523	9.6	6.2676	0.043551	sALS ‐ sFTLD‐TDP, sALS ‐ c9FTLD
	TG(18:1/18:1/18:1)^a^	Positive	907.7709	10.2	7.1797	0.027603	sALS ‐ sFTLD‐TDP
	TG(18:1/18:1/18:2)^a^	Positive	900.8005	10	8.618	0.013447	sALS ‐ sFTLD‐TDP, sALS ‐ c9FTLD
Glycerophospholipids	PC(18:1/20:4)^a^	Positive	808.5845	7	7.763	0.02062	sALS ‐ c9FTLD, sFTLD‐TDP ‐ c9FTLD
Sphingolipids	N,N‐Dimethylsphingosine^c^	Positive	328.3157	3.8	6.7079	0.034946	sALS ‐ c9FTLD, sFTLD‐TDP ‐ c9FTLD
	SM(d33:0)^b^	Positive	689.555	7.2	8.0711	0.017676	sALS ‐ c9FTLD
Sterol lipids	CE(20:1)^a^	Positive	696.6463	8.5	9.5299	0.0085233	sALS ‐ c9FTLD, sFTLD‐TDP ‐ c9FTLD
Unknown	108.0937_0.8405525	Positive	109.0937	0.8	6.0373	0.048868	sALS ‐ sFTLD‐TDP, sALS ‐ c9FTLD
	1145.327_7.909675	Positive	1146.327	7.9	8.0582	0.017791	sALS ‐ sFTLD‐TDP, sFTLD‐TDP ‐ c9FTLD
	1423.392_10.11581	Positive	1424.392	10.1	10.714	0.0047144	sALS ‐ sFTLD‐TDP, sALS ‐ c9FTLD
	224.0327_0.9230345	Negative	223.0327	0.9	11.061	0.0039639	sALS ‐ sFTLD‐TDP, sFTLD‐TDP ‐ c9FTLD
	273.029_1.499844	Positive	274.029	1.5	10.672	0.0048154	sALS ‐ sFTLD‐TDP, sFTLD‐TDP ‐ c9FTLD
	282.2528_3.68679	Negative	281.2528	3.7	5.9962	0.049882	sALS ‐ c9FTLD, sFTLD‐TDP ‐ c9FTLD
	320.3433_3.320781	Positive	321.3433	3.3	6.6419	0.036119	sALS ‐ sFTLD‐TDP, sALS ‐ c9FTLD
	354.2074_0.9234119	Negative	353.2074	0.9	8.5586	0.013853	sALS ‐ sFTLD‐TDP
	666.6668_6.717138	Positive	667.6668	6.7	6.8608	0.032374	sALS ‐ c9FTLD
	738.5654_7.707858	Positive	739.5654	7.7	6.1141	0.047025	sALS ‐ c9FTLD, sFTLD‐TDP ‐ c9FTLD
	773.5415_7.0351	Positive	774.5415	7	9.2808	0.009654	sALS ‐ c9FTLD
	842.8146_10.21985	Positive	843.8146	10.2	9.1588	0.010261	sALS ‐ sFTLD‐TDP, sFTLD‐TDP ‐ c9FTLD
	868.1839_6.287742	Negative	867.1839	6.3	7.2157	0.027109	sALS ‐ sFTLD‐TDP, sFTLD‐TDP ‐ c9FTLD

Significant molecular features Kruskal–Wallis test with *p* value <0.05 are shown as LC‐ESI ionisation mode (P for positive and N for negative) followed by neutral mass and retention time. Potential identities based on MS1 and MS2 data for each feature are found lipidomic feature. Unidentified compounds are labelled as neutral mass and retention time. Ether phospholipids are highlighted in bold.

Abbreviations: CE, cholesterol ester; DG, diacylglycerol; FDR, false discovery rate; mz value, mass‐to‐charge ratio; PC, phosphocholine; SM, sphingomyelin; TG, triacylglycerol.

^a^ Represents confirmation by data‐dependent MS2.

^b^ Represents confirmation by data‐dependent MS2 by class.

^c^ Represents confirmation by MS1 exact mass and retention time.

### Important features associated with different phenotypes

To identify the differential lipid molecules in each disorder, we performed a Kruskal–Wallis test with a post hoc comparison using Dunn's test on frontal cortex region 8 samples from sALS, sFTLD‐TDP, c9FTLD and controls. The differential molecules are listed in Table 5. Globally, phospholipid species (mainly PC) presented the greatest differences between ALS (lower) and CTL (higher) groups. Interestingly, FAHFA (42:3) was decreased in c9FTLD and ALS compared with the control group. Furthermore, we found MG(20:1), CE(20:1) and DG(16:0/22:6) to be elevated in sFTLD‐TDP and c9FTLD compared to the control group. PC(36:3) was down‐regulated in ALS and sFTLD‐TDP compared to controls. PC(18:1/20:4) was down‐regulated in sFTLD‐TDP compared to CTL and c9FTLD. Sphinganine was down‐regulated in ALS and sFTLD‐TDP compared to control samples (Figure 3E).

**TABLE 5:** Lipidomic features associated with neurological disease of the TDP‐43 spectrum

Lipid category	Lipidomic feature	Ionisation	mz value	Retention time	chi squared	*p* value	post hoc
Fatty acyls	FAHFA(42:3)^b^	Negative	643.5684	9.1	10.955	0.01197	sALS ‐ CTL, CTL ‐ c9FTLD, sFTLD‐TDP ‐ c9FTLD
Glycerolipids	DG(16:0/22:6)^a^	Positive	640.5837	7.5	11.308	0.010173	CTL ‐ sFTLD‐TDP, CTL ‐ c9FTLD, sFTLD‐TDP ‐ c9FTLD
	MG(20:1)^c^	Positive	402.3529	3.1	8.9668	0.029736	CTL ‐ sFTLD‐TDP, CTL ‐ c9FTLD
	TG(18:0/18:1/18:1)^a^	Positive	904.8296	10.3	9.3931	0.024496	sALS ‐ sFTLD‐TDP, sFTLD‐TDP ‐ c9FTLD
	TG(18:1/18:1/18:2)^a^	Positive	900.8005	10	8.1153	0.043688	sALS ‐ sFTLD‐TDP, CTL ‐ sFTLD‐TDP
Glycerophospholipids	PC(16:0/20:4)^a^	Positive	782.5691	6.9	8.3459	0.039379	sALS ‐ CTL, CTL ‐ sFTLD‐TDP
	PC(16:0/22:6)^a^	Positive	806.5692	6.8	9.1774	0.027023	sALS ‐ CTL, sALS ‐ c9FTLD, CTL ‐ sFTLD‐TDP
	PC(18:1/20:4)^a^	Positive	808.5845	7	9.7619	0.020703	sALS ‐ CTL, sALS ‐ c9FTLD, CTL ‐ sFTLD‐TDP, sFTLD‐TDP ‐ c9FTLD
	PC(36:3)^b^	Positive	784.5814	7.2	8.278	0.040603	sALS ‐ CTL, sALS ‐ c9FTLD
	PC(44:5)^b^	Positive	846.6285	7.7	8.8661	0.031125	sALS ‐ CTL, sALS ‐ sFTLD‐TDP, CTL ‐ sFTLD‐TDP
	PE(O‐37:1)^b^	Positive	746.5681	7.7	8.1337	0.043328	sALS ‐ CTL, sALS ‐ c9FTLD
	PE(P−16:0/22:6)^a^	Positive	748.5247	7	8.7502	0.032804	sALS ‐ CTL, sALS ‐ c9FTLD, CTL ‐ sFTLD‐TDP
	PE‐NMe(44:12)^c^	Negative	830.5276	7.2	8.0631	0.044726	CTL ‐ c9FTLD, sFTLD‐TDP ‐ c9FTLD
Sphingolipids	SM(d20:5/16:1)^a^	Positive	721.5057	7.8	9.4698	0.023655	sALS ‐ CTL, sALS ‐ c9FTLD, CTL ‐ sFTLD‐TDP
	SM(d33:0)^b^	Positive	689.555	7.2	9.1091	0.027875	CTL ‐ sFTLD‐TDP, CTL ‐ c9FTLD
	Sphinganine^c^	Positive	284.2891	4.4	7.8587	0.049024	sALS ‐ CTL, CTL ‐ c9FTLD
Sterol lipids	CE(20:1)^a^	Positive	696.6463	8.5	11.753	0.0082796	CTL ‐ sFTLD‐TDP, CTL ‐ c9FTLD, sFTLD‐TDP ‐ c9FTLD
Unknown	1145.327_7.909675	Positive	1146.327	7.9	13.69	0.0033584	sALS ‐ CTL, sALS ‐ sFTLD‐TDP, CTL ‐ c9FTLD, sFTLD‐TDP ‐ c9FTLD
	1423.392_10.11581	Positive	1424.392	10.1	11.061	0.011402	sALS ‐ CTL, sALS ‐ sFTLD‐TDP, CTL ‐ sFTLD‐TDP
	224.0327_0.9230345	Negative	223.0327	0.9	11.841	0.0079478	sALS ‐ sFTLD‐TDP, sALS ‐ c9FTLD, sFTLD‐TDP ‐ c9FTLD
	224.19_1.288949	Positive	225.19	1.3	9.4867	0.023473	sALS ‐ CTL, sALS ‐ c9FTLD, CTL ‐ c9FTLD
	273.029_1.499844	Positive	274.029	1.5	15.853	0.0012154	sALS ‐ CTL, sALS ‐ sFTLD‐TDP, sALS ‐ c9FTLD, sFTLD‐TDP ‐ c9FTLD
	320.3433_3.320781	Positive	321.3433	3.3	8.6829	0.033819	sALS ‐ CTL, sALS ‐ sFTLD‐TDP, CTL ‐ sFTLD‐TDP
	348.375_3.32256	Positive	349.375	3.3	8.86	0.031212	sALS ‐ CTL, sALS ‐ c9FTLD, CTL ‐ c9FTLD
	422.4066_7.569828	Negative	421.4066	7.6	8.9619	0.029801	sALS ‐ CTL, sALS ‐ c9FTLD, CTL ‐ sFTLD‐TDP, sFTLD‐TDP ‐ c9FTLD
	598.5024_8.868424	Positive	599.5024	8.9	7.9038	0.048042	sALS ‐ CTL, sALS ‐ c9FTLD
	602.531_8.221487	Positive	603.531	8.2	9.6105	0.022185	sALS ‐ CTL, sALS ‐ c9FTLD, CTL ‐ c9FTLD
	648.6568_6.103854	Positive	649.6568	6.1	9.563	0.022671	sALS ‐ CTL, CTL ‐ c9FTLD, sFTLD‐TDP ‐ c9FTLD
	666.6668_6.717138	Positive	667.6668	6.7	11.872	0.0078361	sALS ‐ CTL, sALS ‐ c9FTLD, CTL ‐ sFTLD‐TDP
	667.059_0.9157435	Negative	666.059	0.9	8.1549	0.042917	sALS ‐ CTL, sALS ‐ sFTLD‐TDP, CTL ‐ sFTLD‐TDP
	703.5196_6.254575	Positive	704.5196	6.3	9.1774	0.027023	sALS ‐ CTL, sALS ‐ c9FTLD, CTL ‐ sFTLD‐TDP
	773.5415_7.0351	Positive	774.5415	7	10.63	0.013903	sALS ‐ CTL, CTL ‐ sFTLD‐TDP,
	780.1863_5.702648	Positive	781.1863	5.7	9.9159	0.019295	sALS ‐ CTL, sALS ‐ c9FTLD, CTL ‐ c9FTLD, sFTLD‐TDP ‐ c9FTLD
	842.8146_10.21985	Positive	843.8146	10.2	8.0448	0.045095	sALS ‐ sFTLD‐TDP, sALS ‐ c9FTLD, sFTLD‐TDP ‐ c9FTLD
	868.1839_6.287742	Negative	867.1839	6.3	9.5946	0.022346	sALS ‐ CTL, sALS ‐ sFTLD‐TDP, CTL ‐ c9FTLD, sFTLD‐TDP ‐ c9FTLD
	981.2558_8.147829	Positive	982.2558	8.1	9.6517	0.021772	sALS ‐ sFTLD‐TDP, sALS ‐ c9FTLD, CTL ‐ c9FTLD

Significant molecular features Kruskal–Wallis test with *p* value <0.05 are shown as LC‐ESI ionisation mode (P for positive and N for negative) followed by neutral mass and retention time. Potential identities based on MS1 and MS2 data for each feature are found lipidomic feature. Unidentified compounds are labelled as neutral mass and retention time. Ether phospholipids are highlighted in bold.

Abbreviations: CE, cholesterol ester; DG, diacylglycerol; FDR, false discovery rate; MG, monoacylglycerol; mz value, mass‐to‐charge ratio; PC, phosphocholine; PE, phophoethanolamine; SM, sphingomyelin; TG, triacylglycerol.

^a^ Represents confirmation by data‐dependent MS2.

^b^ Represents confirmation by data‐dependent MS2 by class.

^c^ Represents confirmation by MS1 exact mass and retention time.

## DISCUSSION

ALS and FTLD are two neurodegenerative disorders that share a number of genetic, pathological and clinical features. One shared molecular trait is the accumulation of pathological protein aggregates including, among others, the protein TAR DNA‐binding protein 43 kDa (TDP‐43).

Previous reports on altered levels of lipids in the central nervous system and peripheral systems in ageing [59, 71] and neurodegenerative disorders [59, 61, 72, 73, 74, 75, 76] led us to hypothesise that impaired peroxisomal function contributes to the progression of neurodegeneration in TDP‐43 proteinopathies. Peroxisomes harbour a variety of enzymes, which either serve to catalyse a single chemical reaction or cooperate with other peroxisomal enzymes in a series of coupled reactions constituting a complete metabolic pathway [11]. A prominent example of these metabolic pathways that links lipid metabolism and peroxisome functioning is the metabolism of diverse fatty acids by α/β‐oxidation, bile acids, docosahexaenoic acid and ether lipid biosynthesis [77]. The present observations show altered gene expression profiles of different components involved in peroxisomal machinery and lipid metabolism that require peroxisomal activity in frontal cortex area 8 of post‐mortem samples of different TDP‐43 proteinopathies.

Our transcriptomic data revealed slight changes in the expression profiles of the studied peroxisomal genes. Gene expression alterations were mainly in genes in peroxisome biogenesis and β‐oxidation, fatty acid metabolism and acylcarnitine biosynthesis mainly found in FTLD cases. Changes in two of six genes linked to primary bile acid biosynthesis do not match with any pattern associated with ALS‐FTLD‐TDP43 proteinopathy. This suggests that the disparate alteration in the biosynthesis of primary bile acids does not have a definite role in FTLD‐TDP43.

In agreement with the slight transcriptomic alterations, no changes in metabolites of bile acid metabolism or alpha/beta fatty acid oxidation were detected with our lipidomic approach, suggesting that the changes in gene expression are insufficient to induce changes at the level of metabolite concentrations, which are maintained within a physiological range. Reinforcing this idea, transcriptomic changes in fatty acid metabolism do not affect the fatty acid profiles of any of the TDP‐43 proteinopathies, suggesting that the activity of the operating machinery, despite being hampered, is sufficient to sustain the optimal fatty acid profile needed to support neuronal survival and function. Thus, fatty acid profile maintains an ACL of about 18 carbon atoms, and a relative distribution between saturated (SFA) and unsaturated (UFA, being UFA = PUFA series n‐3+PUFA series n‐6) fatty acids of around 45:55, with MUFAs being the predominant UFAs, and AA and DHA the main PUFAs, as previously described in healthy adult frontal cortex [59]. Interestingly, the lack of changes in very long chain saturated fatty acids (VLCFA) 22:0 and 24:0 reinforces the idea that the light peroxisomal dysfunction in gene expression associated with TDP‐43 proteinopathies is not sufficient to affect VLCFA content, in contrast to other neuropathological conditions primary of peroxisomal origin such as X‐adrenoleukodystrophy, or even Alzheimer's disease [39, 78].

A sustained fatty acid profile does not exclude, however, potential changes in the content of lipid species that can be uncovered with a lipidomics approach. An adult human brain contains the largest amount and diversity of lipids (in terms of classes and molecular species) including glycerolipids, glycerophospholipids, sphingolipids and cholesterol. Glycerophospholipids are the major phospholipid components ubiquitously found in neural cell membranes [59, 79, 80]. In the human brain, phospholipids constitute 4.2% of the wet weight of the grey matter [79, 81, 82]. Phosphatidic acid occurs in low concentrations in brain (about 2% of total phospholipids). The predominant form of choline phosphoglycerides is PC (32.8%), with PC (16:0/18:1) being the major molecular species; the choline plasmalogen (PC(P‐)) and the alkyl analogue (PC(O‐)) account for only about 2% of total choline phosphoglycerides. Ethanolamine phosphoglycerides are quantitatively the major phospholipid in human brain (35.6%), and the predominant form is the ethanolamine plasmalogen (PE(P‐)), accounting for 50–60% of the ethanolamine phosphoglyceride lipid class in the whole human brain [83]. The alkylacyl analogue content is relatively low (3–7% of the ethanolamine phosphoglyceride class), whereas PE makes up the remaining amount of ethanolamine phosphoglycerides. Their total fatty acid composition indicates a large content of PUFAs, mainly 20:4n6 and 22:6n3 at position‐2 of sn‐glycerol, with position‐1 occupied primarily by 16:0, 18:0 and 18:1 groups in grey matter [83]. The concentration of serine phosphoglycerides is about 16.6%, which is particularly rich in 22:6; and inositol phosphoglycerides account for about 2.6% of the total phospholipids in the human brain [84, 85]. The brain contains the highest concentrations of phosphoinositides among animal tissues. Finally, 0.2% of phospholipids are present as diphosphatidylglycerol in the human brain. Sphingolipids are a complex lipid group, derived from N‐acylsphingosine (ceramide), which occurs in large concentrations in the human brain. This group of lipids consists of sphingomyelin, cerebrosides, sulfatides and gangliosides. Sphingomyelin (N‐acylsphingosine‐1‐phosphocholine) accounts for about 14.8% of the phospholipid content of the human brain.

Our lipidomics approach shows that the ALS‐FTLD‐TDP43 proteinopathy spectrum is associated with minor but significant changes in lipidomic profile, based on the fact that only 63 of 1119 molecular species (5.6%) were significantly different. Interestingly, this lipidomic pattern defines the ALS‐FTLD‐TDP43 proteinopathy spectrum; and with the exception of one molecular species (DG(36:1)), all lipid species (62 out of 63) were decreased. Notably, among the molecules recorded based on exact mass, retention time, and/or MSMS spectrum, no molecular species belong to the phosphatidic acid, phosphatidylserine or phosphatidylinositol class. The differentially identified lipid species belong mainly to glycerophospholipids (PC and PE, and their plasmalogen forms) and sphingolipid lipid families.

Glycerophospholipids are important building blocks of cell membranes that provide an optimal environment for protein interactions, trafficking and function. In the ageing process and in the pathological context of neurodegeneration, decreased brain phospholipid levels and alterations in brain phospholipid metabolism appear, as observed in brain post‐mortem tissue, CSF and blood [86]. This study demonstrates a down‐regulation in PC and PE levels in the ALS‐FTLD spectrum, suggesting alterations in the architecture of the neural cells [87, 88]. In addition, PC is an important source for the formation of second messengers and lipid mediators [89, 90]. Disturbance of its production interferes with cell proliferation and differentiation, and membrane movement throughout the cell [91]. Furthermore, PE plays essential roles in autophagy, cell division and protein folding, representing a precursor for the synthesis of several protein modifications [88]. In addition, both PC and PE are intermediates in the synthesis of other glycerophospholipid classes [92, 93]. In line with our results, a recent study in cells [94] revealed that TDP‐43‐mediated toxicity induced lower levels of glycerophospholipids (especially glycerophosphocholines) and sphingolipids. Decrease levels of glycerophospholipids were also described in ALS animal models [95]. Globally, our results suggest a minor but crucial disturbance of phospholipid metabolism in TDP‐43 proteinopathies. Decreased levels of glycerophospholipids are consistent with altered cell membranes and altered signal transduction via reduced second messengers at the cell membrane. Since the main cellular alteration in the FC of FTLD (and in sALs) affects neurons, it can be inferred that phospholipid alterations here observed reflect abnormal neuronal membrane lipid composition. Whether this is accompanied by altered protein content, indicating cell membranes as putative subcellular targets in TDP‐43 proteinopathies, will be a subject of future studies.

Ether lipids are a subclass of glycerophospholipids that have two chemical forms: as ‘plasmanyl’ (also termed alkyl ethers and represented by the ‘O‐‘ prefix), and as ‘plasmenyl’ (also termed alkenyl ethers or plasmalogens, and represented by the ‘P‐‘ prefix) [96, 97]. Ether lipids are mostly present as PC and PE species [96]. At the cellular level, ether lipid biosynthesis begins in the peroxisome and is completed in the endoplasmic reticulum [96, 97, 98]. The physiological role of ether lipids is linked to their function as membrane components (fluidity, formation of lipid rafts and a source of second messengers). Other functions in which ether lipids are involved are cholesterol transport, G‐protein‐mediated signal transduction, membrane fusion events, transmembrane protein function and vesicular function [96, 97, 98]. Interestingly, an antioxidant effect has also been assigned to plasmalogens [99]. Available evidence reveals that ether lipids are inversely associated with genetic peroxisomal disorders, and also suggests that they are negatively associated with prevalent disease states such as cancer, cardiovascular diseases and Alzheimer disease, among others [100]. Notably, these pathological conditions share as a common trait, increased oxidative stress, and a potential mechanistic role for plasmalogens. Our study clearly demonstrates down‐regulation of the ether lipid content in frontal cortex area 8 of TDP‐43 proteinopathies. Thus, a reduction in its levels may confer vulnerability against oxidative stress insults potentially contributing to neurodegeneration in these disorders, in addition to acting as a marker of impaired peroxisomal function. Reinforcing this idea, the lack of support at the transcriptional level (no changes were observed in the present work) suggests the existence of alterations at the translational level or, more probably, functional defects mediated by potential oxidative stress conditions. Further studies are needed to obtain a more detailed mechanistic view.

A major category of lipids is the sphingolipids [101] that play a key role in the formation of lipid rafts in cell membranes [102]. The metabolism of sphingolipids is a complex network with ceramide at the core [103]. The result is a wide diversity of lipid species with structural (e.g. sphingomyelins) and bioactive/messenger (e.g. sphingosines, dihydroceramides and ceramides) functions [101]. Sphingolipids regulate membrane physiology (fluidity, geometry, membrane trafficking and clustering of plasma membrane receptors and ion channels, among others) and cell biology (e.g. oxidative stress, apoptosis and cell survival), and they have been seen to be involved in several pathological conditions such as cardiovascular diseases and neurodegeneration [103, 104, 105, 106]. Our lipidomic study showed a down‐regulation of structural sphingolipids such as sphingomyelin, as well as bioactive compounds such as sphinganine and ceramides in TDP‐43 proteinopathies, reinforcing the view that there are alterations in lipid metabolism in these neurodegenerative disorders. Therefore, studies focused on lipid rafts, as analysed in detail in Alzheimer's disease, Parkinson's disease and Dementia with Lewy bodies [107, 108, 109, 110] would be extremely useful to refine possible alterations in these cellular domains in which the main cell to cell interactions take place.

Another observation that argues for a peroxisomal dysfunction in TDP‐43 proteinopathies is the down‐regulation of acylcarnitine. Acylcarnitine is a metabolite that plays a relevant role in enabling long‐chain fatty acid exchanges between peroxisomes and mitochondria for beta‐oxidation [111]. Surprisingly, the down‐regulation of acylcarnitines does not seem to be mediated by defects at the transcriptional level. In fact, the contrary is observed, with an increase in mRNA content of the main components of the biosynthesis pathway. Therefore, it is plausible to hypothesise that this apparent contradiction between phenotype and genotype is caused by translational alterations or, analogously to the postulated for plasmalogens biosynthesis, by functional defects at the protein level leading to a reduced peroxisomal biosynthestic capacity. Further studies are, however, also needed to consolidate these new ideas.

Finally, a special mention should be made of the detection of two branched fatty acid esters of hydroxy fatty acids (FAHFAs) for the first time, to our knowledge, in human brain. Although to date their biosynthesis and metabolism are not well elucidated, FAHFAs are a novel class of endogenous lipids that present beneficial effects on glucose homeostasis and anti‐inflammatory activities. Interestingly, there is a possible link between endogenous FAHFA levels and nuclear factor erythroid 2‐related factor 2 (Nrf2), which is involved in cell antioxidant defences, although the mechanisms of this are unknown [112]. Considering the down‐regulation of these compounds in TDP‐43 proteinopathies, it may be suggested that there is impairment of glucose homeostasis, inflammation and oxidative stress in frontal cortex area 8 in ALS‐FTLD‐TDP cases.

Globally, we have demonstrated minor changes in peroxisome‐related gene expression that is mostly involved in lipid metabolism with phenotypic changes affecting ether lipids and acylcarnitine, whereas fatty acid metabolism seems to be preserved in the human frontal cortex in the ALS‐FTLD‐TDP43 proteinopathy spectrum. Additional changes in glycerophospholipids, sphingolipids and specific fatty acid species like FAHFA suggest a more profound impact of these proteinopathies in lipid metabolism with possible consequences on abnormal neuronal membrane composition and impaired cell signalling at the membranes.

At present, little is known about the role of specific lipid species in TDP‐43 proteinopathies and associated neurodegeneration. The development of cell lines and animal models with defective lipid metabolism may help to clarify the role of these lipid species in TDP‐43 proteinopathies thus facilitate better understanding of the mechanisms responsible for neurodegeneration and allowing better diagnosis and treatment.

## CONFLICT OF INTEREST

The authors declare that the research was conducted in the absence of any commercial or financial relationships that could be construed as potential conflicts of interest.

### Peer Review

The peer review history for this article is available at https://publons.com/publon/10.1111/nan.12681.

## Supporting information

Table S1

## Data Availability

The data that support the findings of this study are available from the corresponding author upon request.
