# Supplementary material for: Lipid alterations in human frontal cortex in ALS‐FTLD‐TDP43 proteinopathy spectrum are partly related to peroxisome impairment
Source: Neuropathol Appl Neurobiol. 2021 Jan 12;47(4):544–63. doi: 10.1111/nan.12681 (PMC8248144; doi:10.1111/nan.12681)
Supplement: Supplementary file 1 — Table S1 [file NAN-47-544-s001.docx]

**Supplementary table 1:** **Gene symbols and Taqman probes used in frontal cortex area 8, including normalization GUS-β for normalization.**

| **Gene** | **Full name [Why are these capitalized?]** | **Taqman probe** |
| --- | --- | --- |
| *ABCD1* | *ATP Binding Cassette Subfamily D Member 1* | Hs00163610_m1 |
| *ABCD2* | *ATP Binding Cassette Subfamily D Member 2* | Hs00193054_m1 |
| *ABCD3* | *ATP Binding Cassette Subfamily D Member 3* | Hs00161065_m1 |
| *ACAA1* | *Acetyl-CoA Acyltransferase 1* | Hs01576070_m1 |
| *ACACA* | *Acetyl-CoA Carboxylase Alpha* | Hs00167385_m1 |
| *ACOT1* | *Acyl-CoA Thioesterase 1* | Hs02598489_mH |
| *ACOX1* | *Peroxisomal Acyl-Coenzyme A Oxidase 1* | Hs01074241_m1 |
| *ACOX2* | *Peroxisomal Acyl-Coenzyme A Oxidase 2* | Hs00185873_m1 |
| *ACOX3* | *Peroxisomal Acyl-Coenzyme A Oxidase 3* | Hs01089970_m1 |
| *ACSL1* | *Acyl-CoA Synthetase Long Chain Family Member 1* | Hs00242530_m1 |
| *ACSL3* | *Acyl-CoA Synthetase Long Chain Family Member 3* | Hs00244853_m1 |
| *ACSL4* | *Acyl-CoA Synthetase Long Chain Family Member 4* | Hs01547083_m1 |
| *ACSL5* | *Acyl-CoA Synthetase Long Chain Family Member 5* | Hs01061754_m1 |
| *ACSL6* | *Acyl-CoA Synthetase Long Chain Family Member 6* | Hs00922295_m1 |
| *AGPS* | *Alkyldihydroxyacetonephosphate Synthase, Peroxisomal* | Hs01056969_m1 |
| *CAT* | *Catalase* | Hs00156308_m1 |
| *CH25H* | *Cholesterol 25-Hydroxylase* | Hs02379634_s1 |
| *CRAT* | *Carnitine O-acetyltransferase* | Hs00912963_m1 |
| *CROT* | *Carnitine O-Octanoyltransferase* | Hs00221733_m1 |
| *CYP27A1* | *Cytochrome P450 Family 27 Subfamily A Member 1* | Hs01017992_g1 |
| *CYP39A1* | *Cytochrome P450 Family 39 Subfamily A Member 1* | Hs00213201_m1 |
| *DHAP-AT* | *Glyceronephosphate O-Acyltransferase* | Hs00204517_m1 |
| *DNM1L* | *Dynamin 1 Like* | Hs00247147_m1 |
| *EHHADH* | *Enoyl-CoA Hydratase And 3-Hydroxyacyl CoA Dehydrogenase* | Hs00157347_m1 |
| *ELOVL1* | *Elongation Of Very Long Chain Fatty Acids Protein 1* | Hs00249277_m1 |
| *ELOVL2* | *Elongation Of Very Long Chain Fatty Acids Protein 2* | Hs00214936_m1 |
| *ELOVL4* | *Elongation Of Very Long Chain Fatty Acids Protein 4* | Hs00224122_m1 |
| *ELOVL5* | *Elongation Of Very Long Chain Fatty Acids Protein 5* | Hs01094711_m1 |
| *ELOVL6* | *Elongation Of Very Long Chain Fatty Acids Protein 6* | Hs00225412_m1 |
| *ELOVL7* | *Elongation Of Very Long Chain Fatty Acids Protein 7* | Hs00405151_m1 |
| *FAR1* | *Fatty Acyl-CoA Reductase 1* | Hs00386153_m1 |
| *FASN* | *Fatty Acid Synthase* | Hs00188012_m1 |
| *GPX1* | *Glutathione Peroxidase 1* | Hs02516751_s1 |
| *GUS-β* | *β-glucuronidase* | Hs00939627_m1 |
| *HSD17B4* | *Hydroxysteroid 17-Beta Dehydrogenase 4* | Hs00264973_m1 |
| *HSD3B7* | *Hydroxy-ƌ-5-Steroid Dehydrogenase, 3 β- nd Steroid ƌ-Isomerase 7* | Hs00228639_m1 |
| *PEX14* | *Peroxisomal Biogenesis Factor 14* | Hs00992866_m1 |
| *PPARA* | *Peroxisome Proliferator Activated Receptor Alpha* | Hs00947539_m1 |
| *PPARD* | *Peroxisome Proliferator Activated Receptor Delta* | Hs00606407_m1 |
| *PPARG* | *Peroxisome Proliferator Activated Receptor Gamma* | Hs01115513_m1 |
| *PPARGC1A* | *PPARG Coactivator 1 Alpha* | Hs00173304_m1 |
| *SCD1* | *Stearoyl-CoA Desaturase* | Hs01682761_m1 |
| *SCD5* | *Stearoyl-CoA Desaturase 5* | Hs00227692_m1 |
| *SCP2* | *Sterol Carrier Protein 2* | Hs03004033_m1 |
